# Alternative pre-analytic sample handling techniques for glucose measurement in the absence of fluoride tubes in low resource settings

**DOI:** 10.1371/journal.pone.0264432

**Published:** 2022-02-28

**Authors:** Wisdom P. Nakanga, Priscilla Balungi, Anxious J. Niwaha, Beverly M. Shields, Peter Hughes, Rob C. Andrews, Tim J. Mc Donald, Moffat J. Nyirenda, Andrew T. Hattersley

**Affiliations:** 1 National Institute for Health Research (NIHR), Exeter Clinical Research Facility, University of Exeter, Exeter, United Kingdom; 2 Medical Research Council/ Uganda Virus and Research Institute and LSHTM Uganda Research Unit, Entebbe, Uganda; 3 London School of Hygiene and Tropical Medicine (LSHTM), London, United Kingdom; Government College University Faisalabad, Pakistan, PAKISTAN

## Abstract

**Introduction:**

Sodium fluoride (NaF) tubes are the recommended tubes for glucose measurements, but these are expensive, have limited number of uses, and are not always available in low resource settings. Alternative sample handling techniques are thus needed. We compared glucose stability in samples collected in various tubes exposed to different pre-analytical conditions in Uganda.

**Methods:**

Random (non-fasted) blood samples were drawn from nine healthy participants into NaF, Ethylenediaminetetraacetic acid (EDTA), and plain serum tubes. The samples were kept un-centrifuged or centrifuged with plasma or serum pipetted into aliquots, placed in cool box with ice or at room temperature and were stored in a permanent freezer after 0, 2, 6, 12 and 24 hours post blood draw before glucose analysis.

**Results:**

Rapid decline in glucose concentrations was observed when compared to baseline in serum (declined to 64%) and EDTA-plasma (declined to 77%) after 6 hours when samples were un-centrifuged at room temperature whilst NaF-plasma was stable after 24 hours in the same condition. Un-centrifuged EDTA-plasma kept on ice was stable for up to 6 hours but serum was not stable (degraded to 92%) in the same conditions. Early centrifugation prevented glucose decline even at room temperature regardless of the primary tube used with serum, EDTA-plasma and NaF-plasma after 24 hours.

**Conclusion:**

In low resource settings we recommend use of EDTA tubes placed in cool box with ice and analysed within 6 hours as an alternative to NaF tubes. Alternatively, immediate separation of blood with manual hand centrifuges will allow any tube to be used even in remote settings with no electricity.

## Introduction

Glucose measurement is crucial for appropriate diagnosis and management of diabetes. However, obtaining accurate results requires careful sample handling, including correct use of blood collecting tubes, sample processing and timely analysis. The current guidelines recommend use of tubes containing sodium fluoride (NaF) for glucose measurement due to its ability to inhibit ex-vivo glycolysis [1, 2]. These pre-analytical requirements can be a challenge in resource poor settings, such as in many parts of Sub Saharan Africa (SSA) [3, 4]. For example, supply of NaF tubes may be erratic or might be thought uneconomical to use such tubes because they are only suitable for glucose and lactate analysis [5]. In addition, there are delays in sample analysis, commonly due to challenges in transporting samples to laboratories which are typically away from sites of blood collection. This is coupled with problems of inconsistent power supply and outages that are endemic in the region [6, 7]. The question of glucose stability also becomes important because of high ambient temperature in many SSA countries. Previous studies of glucose stability at ‘room temperature’ have used temperatures often far below those that may be experienced in many African situations, and therefore these studies may not apply to the SSA setting.

Alternative pre-analytic sample handling techniques to optimise glucose measurement have been suggested. These include immediate centrifugation and placing samples in ice water for analysis within one-hour post collection [2, 8]. But none of these studies have been in the context of SSA. We therefore set out to assess the effect of different pre-analytical conditions on measured glucose concentration, with a view to identifying alternative pre-analytic sample handling procedures that can provide accurate glucose results in the absence of NaF.

## Methods

Participants with no history of diabetes were invited to the MRC/UVRI and LSHTM Unit laboratory in Uganda for the blood tests.

The study was approved by Uganda Virus Research Institute Research Ethics Committee, Uganda National Council for Science and Technology, and London School of Hygiene and Tropical Medicine Ethics Committee. Written informed consent was obtained from all participants.

**Fig 1:**
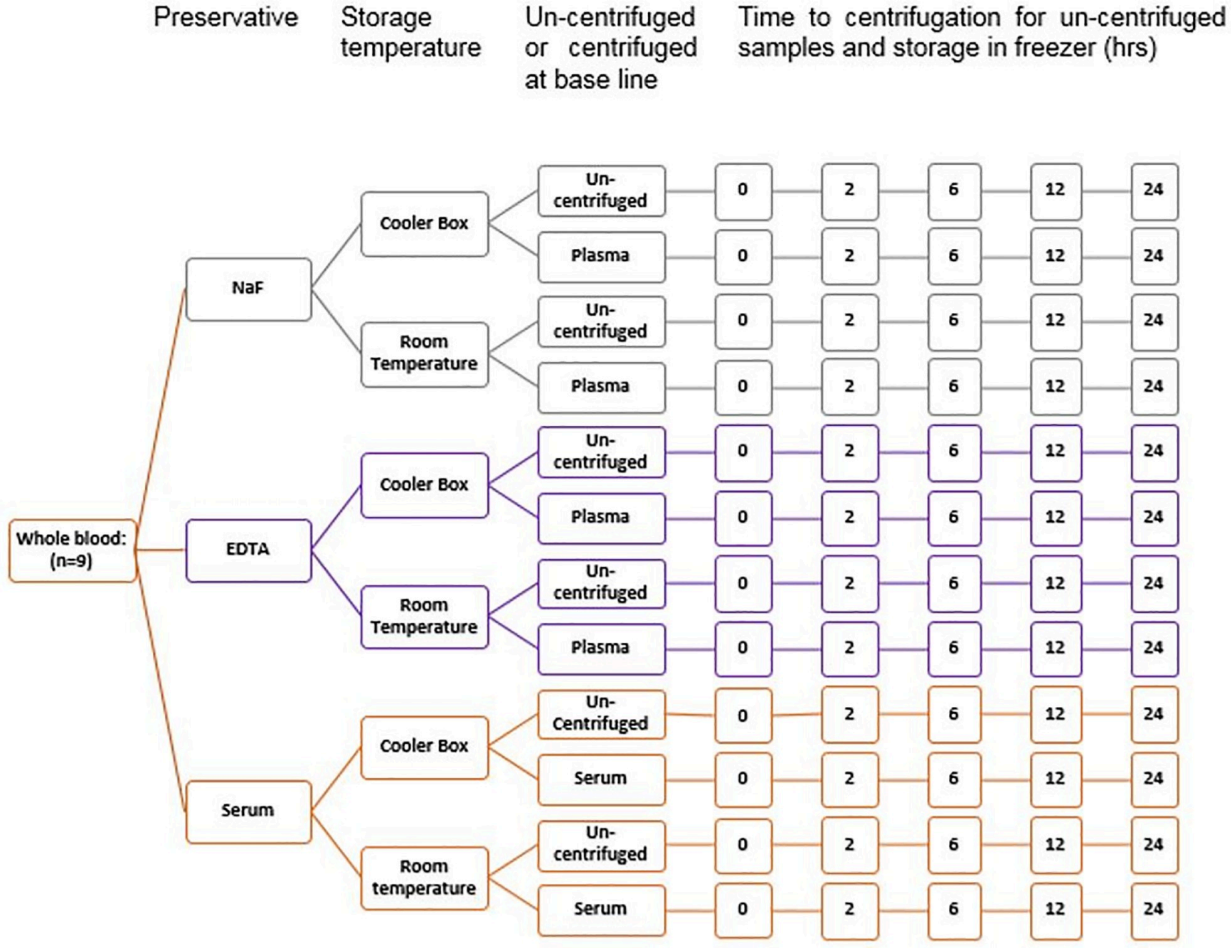
Flow diagram detailing the sample collection protocol for the study over 24 hours. At each time-point the samples were centrifuged for 10 minutes. The supernatant was frozen at -20°C.

60 mls of non-fasted venous blood was taken in the morning (glucose range 4.1–8.0 mmol/L), using an 18 g needle and 20 ml syringes, and allocated into 20 NaF, 20 Ethylenediaminetetraacetic (EDTA) and 20 plain tubes (Fig 1). 4 NaF, 4 EDTA and 4 plain tubes were centrifuged at 3000G for 10 minutes within 30 minutes from being taken and allocated into tubes and stored in a -20°C freezer. These were used as our reference specimens.

8 NaF, 8 EDTA and 8 plain tubes were centrifuged at 3000G for 10 minutes within 30 minutes from being taken and allocated in aliquot tubes such that there were 8 aliquots from the NaF bottles, 8 from the EDTA and 8 from the plain tubes. 4 of each of these were kept at room temperature and 4 in cool box with ice. At time points 2, 6, 12 and 24 hours one aliquot from each tube from the room temperature and cool box were placed in the -20°C freezer. 4 NaF, 4 EDTA and 4 plain tubes were stored at room temperature with 1 of each of these bottles centrifuged, aliquoted and placed in -20°C freezer at 2, 6, 12 and 24 hrs from when the sample was taken. Lastly 4 NaF, 4 EDTA and 4 plain tubes were stored in the cool box with the same procedure followed as that with the specimens stored at room temperature.

The samples were analysed as a batch at the MRC/UVRI and LSHTM Clinical Diagnostic Laboratory in Entebbe, Uganda (coefficient of variation for glucose measurement 1%) using the glucose oxidase method on the Cobas 6000 analyser (Roche/Hitachi, Tokyo, Japan). We compare results of samples kept in four different preanalytic conditions: (1) un-centrifuged kept at room temperature, (2) un-centrifuged kept in cool box with ice, (3) centrifuged kept at room temperature (4) centrifuged kept in cool box with ice.

### Statistical analysis

The baseline glucose concentrations of all the samples was compared using analysis of variance. Results are presented as mean percentage change from baseline (sample centrifuged, separated and frozen at time point zero). The difference between the baseline and glucose concentration at different time points was assessed by Wilcoxon signed-rank test. We considered samples to be the same if there was a mean change of less than 10 percent from baseline and P value of greater than 0.05.

## Results

Nine people (seven males, two females, mean age 30.8, range: 24–50) volunteered to take part in the experiment and attended in September 2019 for their blood test The baseline glucose levels from NaF, EDTA and serum tubes were similar 5.4mmol/L, 5.4mmol/L and 5.2mmol/L respectively (P = 0.77). The mean ambient room temperatures after 6 and 24 hours was 28°C and 25°C which were higher than their corresponding temperatures in the cool box, 6°C and 15°C.

### Rapid decline in glucose if left un-centrifuged at room temperature unless in NaF

**Fig 2:**
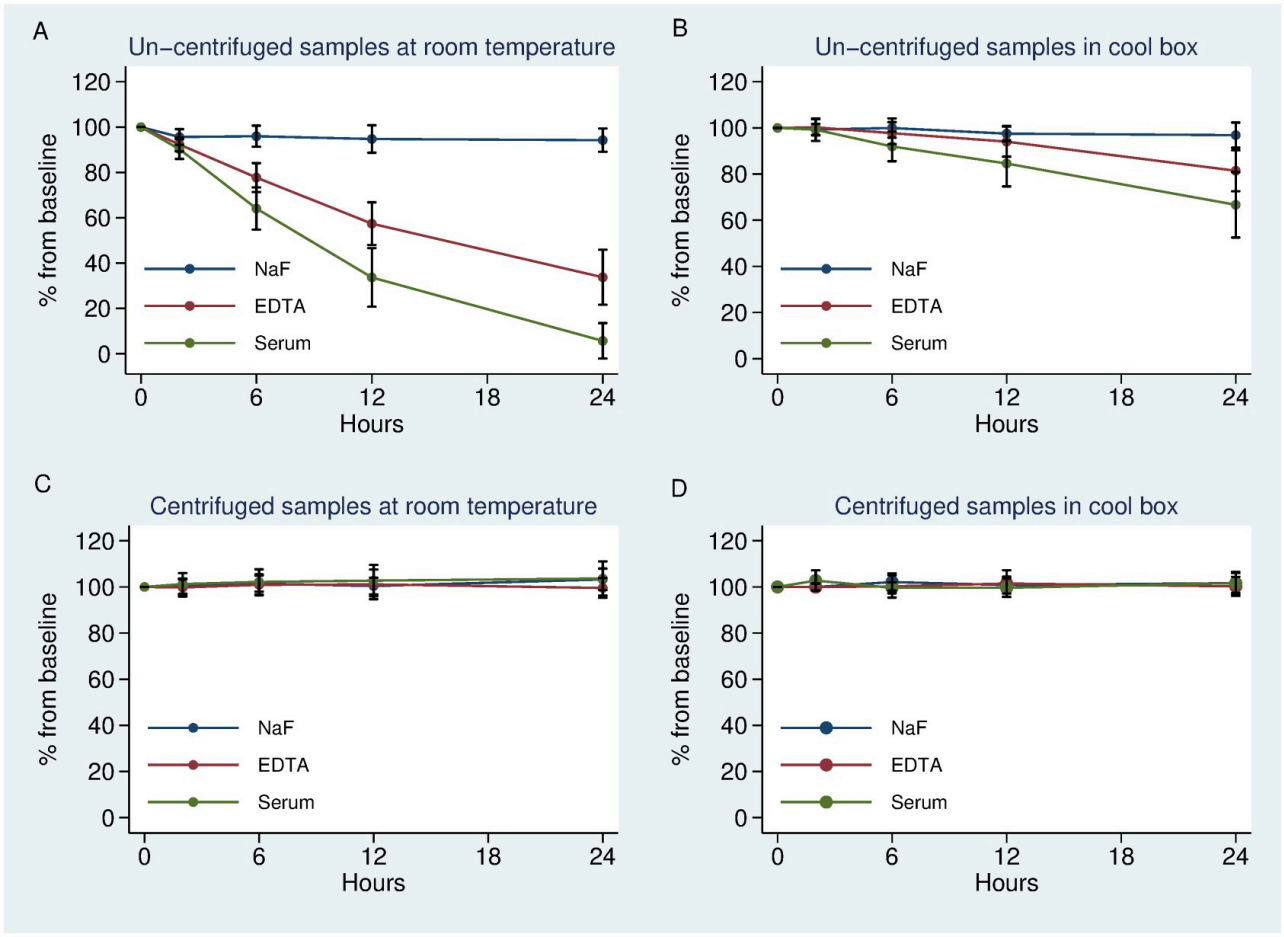
The stability over time for each of the scenarios investigated. Mean percentage change from baseline shown ± 95% confidence interval (CI).

Un-centrifuged plasma and serum from EDTA and Serum tubes decreased to 77% (P = 0.004) and 64% (P = 0.004) respectively from baseline after 6 hours and by 34% (P = 0.004) and 6% (P = 0.004) respectively after 24 hours (Fig 2A). In contrast samples kept un-centrifuged in NaF tube had declined to 94% (P = 0.18) at 6 hours and there after remained stable up to 24hours.

### Un-centrifuged EDTA plasma but not serum was stable for 6hours if kept on ice at 6°C

Un-centrifuged NaF and EDTA plasma that were kept in cool box was stable after 6 hours 100% (P = 1) and 97% (P = 0.18) respectively (Fig 2B). Serum from plain tubes that was stored in a cool box was stable after 2 hours 99% (P = 1) but decreased to 92% (P = 0.004) from baseline 6 hours after blood draw.

### Early centrifugation prevented glucose decline even at room temperature

All NaF, EDTA and serum samples that were centrifuged immediately after collection, and plasma or serum separated from cells and placed in cool box with ice were stable at 24 hours (Fig 2D). Plasma and serum samples centrifuged immediately after collection were also stable at room temperature after 24 hours regardless of the primary tube used, NaF 103% (P = 0.17), EDTA 100%(p = 0.51) and plain tube 103% (P = 0.5) (Fig 2C).

## Discussion

In resource limited settings, where NaF tubes are unavailable, glucose can be accurately measured with appropriate pre-analytical handling. We have shown that if serum or plasma from EDTA and plain tubes is separated within 30 minutes it can be stored at room temperature and used for up to 24 hours to accurately measure glucose. In addition, an EDTA specimen if stored on ice can be separated up to 6 hours after being taken and still provide an accurate measure of glucose. This offers potential viable alternative techniques for blood collection and handling that can be utilized in clinical and research settings in SSA.

Glucose falls rapidly in un-centrifuged samples kept at room temperature, even by two hours, in the absence of a glycolytic inhibitor, fluoride [9]. This emphasises the need for appropriate sample handling techniques, for example the use of cooling agent or rapid centrifugation in scenarios where delay in analysis may occur. However even when NaF tubes were used, a small decline was observed in the first two hours with stability achieved thereafter up to 24 hours. This is because the fluoride acts by inhibiting enolase enzyme which occurs further down the glycolytic pathway and does not inhibit the enzymes upstream of enolase which continue to metabolize glucose 6 phosphate [10].

The pragmatic approach of not replacing the ice blocks in the cool box, resulted in stability of EDTA-plasma glucose for up to six hours. The decreased temperature results in corresponding decrease in cellular metabolism leading to preservation of glucose [11]. However we found that un-centrifuged serum was not stable even on ice. This has also been found in other studies [12]. We recommend that EDTA tubes can be used in sample collection for glucose analysis if samples are places on ice immediately after blood draw and analysed or centrifuged within 6 hours.

Glucose was best preserved when the samples were centrifuged immediately even in serum tubes. The use of serum could enable many biochemical measures to be analysed in a single sample [13]. Innovations such as 3D printed hand held centrifuges could offer solutions to the obvious challenges with advocating for immediate separation of blood in remote areas where conventional centrifuges and electricity are not readily available [14]. The glucose concentration in the EDTA-plasma was slightly lower than in the comparable tubes which could be attributed to the presence of platelets which also undergo glucose metabolism [7].

Illustrated weakness of the study is the limited range of the glucose results. It is not clear from our study how the EDTA tubes will perform in very high or low glucose values. Another drawback is that we had a limited sample size and therefore were unable to observe differences, for example in the baseline plasma and serum glucose which has been reported in other studies [15]. Lastly, other blood parameters that are known to alter glucose concentration, for example white blood cells and platelets were not measured. However this study is amongst the first to assess the impact of several conditions on glucose results on the same samples in a low resource setting in a region with high ambient temperatures.

## Conclusion

NaF tubes remains the preferred collection tubes for glucose measurement in research and clinical setting. In absence of these, we recommend the use of EDTA tubes provided they are immediately placed on ice and analysed or centrifuged within 6 hours. If facilities for immediate centrifugation are available, then any collection tube can be used for glucose measurement and samples can be placed at room temperature.

## Supporting information

S1 Data (CSV)
